# Life-Threatening Abnormal Behavior Incidence in 10–19 Year Old Patients Administered Neuraminidase Inhibitors

**DOI:** 10.1371/journal.pone.0129712

**Published:** 2015-07-01

**Authors:** Yuuki Nakamura, Tamie Sugawara, Yasushi Ohkusa, Kiyosu Taniguchi, Chiaki Miyazaki, Mariko Momoi, Nobuhiko Okabe

**Affiliations:** 1 Graduate School of Pharmacy, Nihon University, Funabashi, Chiba, Japan; 2 Infectious Disease Surveillance Center, National Institute of Infectious Diseases, Shinjuku, Tokyo, Japan; 3 National Hospital Organization Mie National Hospital, Tsu, Mie, Japan; 4 Fukuoka Welfare Center for the Disabled, Fukuoka, Fukuoka, Japan; 5 International University of Health and Welfare, Ohtawara, Tochigi, Japan; 6 Kawasaki City Institute for Public Health, Kawasaki, Kanagawa, Japan; Chiba University Center for Forensic Mental Health, JAPAN

## Abstract

Much discussion has surrounded the association between the administration of neuraminidase inhibitors (NI) and severe abnormal behaviors, including sudden running away and jumping from a high place, which can be life-threatening if no one intervenes. Using data on the number of abnormal behaviors and patients who had been prescribed NI in Japan, we calculated the incidence rate of severe abnormal behaviors among influenza patients who had been prescribed NI. Then, we evaluated the relative risk between the four types of NI on severe abnormal behavior. We found no significant difference in the incidence rates of abnormal behavior by the type of NI. Results implicate that the current policy of package inserts, which warn physicians that patients who were administered ANY type of NI might exhibit abnormal behavior, seems to be appropriate.

## Introduction

In February 2007, two Japanese Junior high students who had contracted influenza jumped from a great height and died. At that time, oseltamivir was presumed to cause these events. As a response to these events, the Dear Healthcare Professional Letters of Emergent Safety Communications (the Yellow Letter) published in March 2007 ordered physicians to inhibit the prescription of oseltamivir to 10–19-year-old influenza patients, with a particular exclusion of high-risk patients who have a complication or medical history [1]. Moreover, because the patients who were administrated other neuraminidase inhibitors (NI) also showed abnormal behavior in the report from all outpatient clinics and hospitals throughout Japan [2], mandatory package inserts for NI of all types have warned about abnormal behavior since December 2007 [3–6]. Moreover, the Japanese Ministry of Health, Labour and Welfare (MHLW) has advised caregivers to devote attention to influenza-like illness (ILI) patients irrespective of the administration of any drug since April 2007.

Many studies, mainly conducted in Japan, have examined the relation between abnormal behaviors and the administration of oseltamivir [7–14] or laninamivir [15,16]. Particularly, a survey of abnormal behaviors of influenza patients from all physicians revealed abnormal behaviors of patients who had and had not been administered NI [17].

A few studies have indicated abnormal behaviors of patients who had been administered NI in other countries than Japan, such as the US, China, and European countries [18–20]. Toovey et al. (2012) reported 1,805 neuropsychiatric adverse events (NPAEs) in 1,330 patients who had been receiving oseltamivir, and reported 454 events of delirium and delirium-like events [18]. The US FDA Adverse Event Reporting System received 980 NPAEs associated with oseltamivir [19]. In addition, Jefferson et al. (2014) reviewed clinical report describing some effects of oseltamivir [20]. That review examined 47 psychiatric adverse events occurring in 44 patients taking oseltamivir arms in 23 trials.

Unfortunately, these studies have not investigated the relative risk of life-threatening abnormal behaviors by NI. They only examined oseltamivir or laninamivir, and did not consider other NIs such as zanamivir or peramivir. Because the number of influenza patients taking NI were not presented in the study periods and studied areas in these studies, they failed to identify the incidence rate of life-threatening abnormal behaviors by NI. Additionally, the abnormal behavior in these studies was not well defined; they might include some mild abnormal behaviors which might not affect the patient’s life.

Therefore, we examined incidence rates of the most severe abnormal behaviors of influenza patients who were prescribed four types of NI, and compared these incidence rates to evaluate the relative risk of abnormal behaviors associated with NI. Using the incidence rates, we assessed the association between the type of NI and severe abnormal behaviors. To calculate the incidence rates of patients with severe abnormal behaviors among influenza patients, it is necessary to ascertain the precise number of influenza patients. We used the number of patients who had been prescribed NI from data, which were shown at the Subcommittee on Drug Safety of Committee on Drug Safety in the Pharmaceutical Affairs and Food Sanitation Council of the MHLW (http://www.mhlw.go.jp/stf/shingi/shingi-yakuji.html?tid=127869, in Japanese). The survey includes the estimated number of patients who were prescribed NI during each influenza season, although the details of procedures used for estimating the number of patients were not well explained.

Unfortunately, the data shown at the Subcommittee as mentioned above did not include the estimated number of ILI patients who were not prescribed any NI, even though the survey of abnormal behavior included the case without any NI [2,17]. In other words, we cannot define the control group who were not prescribed any NI so as to evaluate the absolute risk of any NI. Therefore, in this study we focus on the relative risk among types of NI rather than the absolute risk of each NI.

## Materials and Methods

### Data and Study Period

Definitions of abnormal behavior in patients with influenza and the methods for investigation were described in a report of an earlier study [17]. All cases of patients with influenza who presented with severe abnormal behavior were reported from physicians of all outpatient clinics and hospitals throughout Japan, based on the administrative order from the section manager of the Tuberculosis and Infectious Diseases Control Division, MHLW, and the section manager of Safety Division of Pharmaceutical and Food Safety Bureau, MHLW. All reports were made either online or via fax to the Infectious Diseases Surveillance Center, National Institute of Infectious Diseases (NIID).

In this study, ILI was defined as showing all the following symptoms: acute onset, high fever >38°C, upper respiratory symptoms, systemic symptoms including fatigue, or positive results from an influenza rapid diagnosis kit [17]. We defined severe abnormal behavior as active motion behavior which can be life-threatening if no one intervenes, including those such as sudden running away, jumping from a high place, and rampaging involving self-injury. Moreover, we defined sudden running away and jumping from a high place as the most severe abnormal behaviors because they would engender death with a high probability. We specifically focused on the analysis of the most severe abnormal behaviors.

In Japan, the influenza season is defined as the period from the 36th epidemiological week to the 35th week of the following year. We undertook the survey for the abnormal behaviors in 2006/2007 to 2013/2014 season. However, since the approval of laninamivir and peramivir was completed in 2010/2011 season, we did not use data for periods before 2010/2011 season.

The number of patients who were prescribed NI was referred from published information of the Subcommittee on Drug Safety of Committee on Drug Safety in the Pharmaceutical Affairs and Food Sanitation Council of the MHLW (http://www.mhlw.go.jp/stf/shingi/shingi-yakuji.html?tid=127869) [21–24]. Data consists of the estimated numbers of patients prescribed NI by the type and proportion of prescription of NI in each season. We used these data from 2010/2011 to 2013/2014 seasons. We specifically examined 10–19-year-old patients because they were regarded as the patients most affected by NI and data was collected mainly on this group at the Subcommittee.

### Analysis

We used analysis of variance (ANOVA) to assess the difference in the incidence rate of abnormal behaviors by NI. We also evaluated the difference in the incidence rates in any pair of type of NI using a Fisher’s exact test. We adopted 5% as the significance level.

### Ethics

For the investigation of abnormal behavior, because we did not collect any personally identifiable information such as name, address, and date of birth, patient records and information were anonymized and de-identified before analysis. Moreover, we have only counted the number of patients with a certain condition from the collected information. The ethical guidelines for epidemiological research in Japan do not require receipt of informed consent from patients in this case. The studies of abnormal behavior were approved by the NIID committee for ethical consideration: approval numbers were 261, 312, 375, and 462.

## Results

Table 1 presented the estimated number of 10–19-year-old patients who were prescribed NI in each season. The maximum number of all estimated numbers of patients prescribed NI was approximately 2.2 million during the 2010/2011 season. The maximum and minimum numbers of each NI were, respectively, 0.22 and 0.13 million for oseltamivir, 1.01 and 0.62 million for zanamivir, 0.85 and 0.66 million for laninamivir, and 0.04 and 0.02 million for peramivir.

**Table 1:** Estimated number of 10–19-year-old patients prescribed neuraminidase inhibitors each season (million cases).

	Estimated numbers of patients prescribed NI (million cases)				
Season	all	oseltamivir	zanamivir	laninamivir	peramivir
2010/2011	2.20^a)^	0.22^a)^	1.01^h)^	0.66^j)^	0.04^n)^
2011/2012	1.80^b)^	0.16^e)^	0.66^h)^	0.83^k)^	0.03^o)^
2012/2013	1.70^c)^	0.14^f)^	0.62^h)^	0.85^l)^	0.02^p)^
2013/2014	1.60^d)^	0.13^g)^	0.62^i)^	0.85^m)^	0.03^q)^

Note: Each estimated number of patients prescribed NI was referred from published information as follows.

a) Page 3 in [21]

b) Page 5 in [22]

c) Page 5 in [23]

d) Page 3 in [24]

e) Page 6 in [22]

f) Page 6 in [23]

g) Page 4 in [24]

h) Page 8 in [23]

i) Page 5 in [24]

j) Page 8 in [21]

k) Page 10 in [22]

l) Page 10 in [23]

m) Page 7 in [24]

n) Page 7 in [21]

o) Page 9 in [22]

p) Page 9 in [23]

q) Page 6 in [24]

As defined, the season begins in the 36th epidemiological week, ending the 35th week of the following year. Since approval for laninamivir and peramivir was in 2010, we ignored data for periods before 2010.

**Fig 1:**
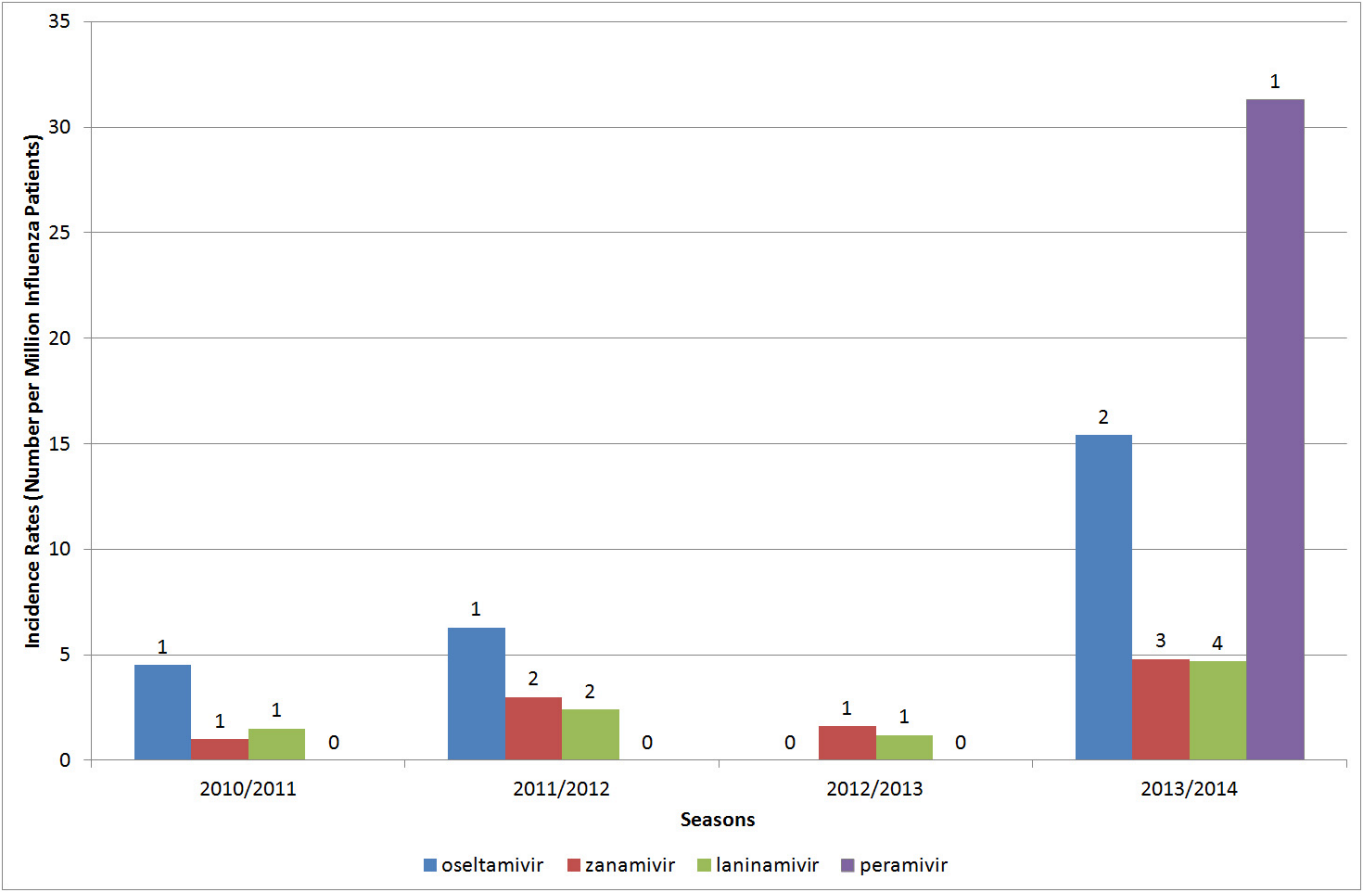
Number of patients (cases) and incidence rates (number per million influenza patients) of the most severe abnormal behaviors of 10–19-year-olds in each season. Since approvals for laninamivir and peramivir were in 2010, we ignored data before 2010. This figure shows the number of patients with the most severe abnormal behaviors of 10–19-year-olds, which are shown on bar charts, and incidence rates in number per million influenza patients, which are shown as bar charts in each season.

Fig 1 presented the number of cases of abnormal behaviors and incidence rates of patients with the most severe abnormal behaviors per million 10–19-year-old patients who were prescribed each type of NI in each season. The numbers of cases of abnormal behaviors for each NI were, respectively, from 0 to 2 for oseltamivir, from 1 to 3 for zanamivir, from 1 to 4 for laninamivir, and from 0 to 1 for peramivir. The highest incidence rates per million influenza patients were, respectively, 15.4 for oseltamivir, 4.8 for zanamivir, 4.7 for laninamivir, and 31.3 for peramivir. In total, during 2010/2011 through 2013/2014 season, incidence per million influenza patients were 6.1 for oseltamivir, 2.4 for zanamivir, 2.5 for laninamivir, and 8.3 for peramivir, respectively.

Regarding the results of ANOVA for NI of all types related to abnormal behaviors of 10–19-year-old from 2010/2011 through 2013/2014, *p*-value and F-statistics were 0.749 and 0.41. Therefore, it presented no significance. Table 2 showed the results of Fisher’s exact tests in any pair of type of NI, and there were no significant differences in any pair of type of NI.

**Table 2:** Results of Fisher’s exact tests for incidence rates of the most severe abnormal behavior among types of NI during 2010/2011 through 2013/2014 season (*p*-value).

	zanamivir	laninamivir	peramivir
oseltamivir	0.126	0.131	0.572
zanamivir		1.000	0.278
laninamivir			0.284

Note: Numbers of patients with the most severe abnormal behavior per million influenza patients of 10–19-year-olds were 6.1 (oseltamivir), 2.4 (zanamivir), 2.5 (laninamivir), 8.3 (peramivir) during 2010/2011 through 2013/2014 season.

## Discussion

We found no significant difference among the incidence rates of the most severe abnormal behaviors by NI. Such a relative risk has never examined before. We focused on the most severe abnormal behaviors as they are the most serious public health concerns. However, focusing on those behaviors necessarily reduced the sample size, and such a small sample might be the reason for our inconclusive results. Although, as mentioned above, the most severe abnormal behaviors were investigated nationwide [17], such behaviors are very rare. Therefore, we cannot expand the number of cases immediately by adding new study fields. Therefore, accumulation of data by continuing the research is expected to be necessary to find a definitive conclusion about differences in risk for the most severe abnormal behavior of influenza patients.

The Dear Healthcare Professional Letters of Emergent Safety Communications related to oseltamivir administration required physicians to refrain from prescribing oseltamivir from 10–19-year-old patients with influenza, exception for high-risk patients. Therefore, 10–19 years old were presumably high-risk patients who were administrated with oseltamivir. Unfortunately, we have no information about complications or medical history related to high risk. Furthermore, we do not know that such a high risk might be particularly associated with abnormal behaviors. High-risk patients might be more prone to abnormal behaviors, but such an association might be hypothetical.

Since we found no significant difference among all types and in any pair of type of NI, we cannot speculate about the relative risk of types of NI. If we took account of multiple-test adjustment, e.g. Bonferroni and/or Holm correction for Fisher’s exact test, the conclusion should not be affected because all *p*-values of any pair of type of NI were less than the significant level. On the other hand, the patients with the most severe abnormal behaviors had been reported in all types of NI. Therefore, we considered that the policy of the package inserts, which warned physicians that patients who were administered any types of NI might exhibit abnormal behavior, is appropriate.

Concerning the number of patients who were prescribed NI, we used the estimated number of patients provided at the Subcommittee on Drug Safety of Committee on Drug Safety in the Pharmaceutical Affairs and Food Sanitation Council of the MHLW. These data were estimated from three databases: Japan Medical Information Research Institute (JMIRI) [25], Japan Medical Data Center (JMDC) [26], and JammNet [27]. The JMIRI data based on prescription claims from 400 panel pharmacies, which account for approximately 0.9% of all pharmacies. The JMDC and JammNet data based on medical and prescription claims for 1 million and 0.5 million population, respectively accounting for approximately 0.8%, and 0.4% of the total population. However, because the data coverage is too small, the sample representativeness of the databases is apparently problematic. Moreover, details of the procedures for estimating the number of patients were not well explained. Therefore, the estimated number of influenza patients used in this study might be questionable as the precise number of patients. One previous study had validated the estimated number of influenza patients in JMDC with Prescription Surveillance (PS) [28]. The estimated number of influenza patients through PS, which accounts of approximately 19.6% of all pharmacies in nationwide, was validated with all electronic medical claims (National Database of Electronic Medical Claims, NDBEMC) [29,30], accounting for about 96.2% of all medical claims. Moreover, all physicians must record a diagnosis on medical claims [31–33]. Therefore, NDBEMC is regarded as the most reliable data source. However, our data of the estimated number of patients with each NI from databases of JMIRI, JMDC, and JammNet, which were published from MHLW, has not been validated by NDBEMC.

## Conclusion

This is the first study that examined the relative risk of the most severe abnormal behaviors among type of NI. The obtained result showed no significant difference among the incidence rates of the most severe abnormal behaviors by NI. However, severe abnormal behaviors with all types of NI had been reported. Therefore, we infer that the policy mandating package inserts in NI of all types, and asking physicians about abnormal behavior, seems to be appropriate. It is important to continue the investigation for abnormal behaviors and accumulate data so as to reach definitely conclusion.

We should note again that the incidence rate of the most severe abnormal behaviors among ILI patients who were not prescribed NI is not available because the denominators were not available in information provided from the Subcommittee. Therefore we cannot evaluate the absolute risk of any NI. This lack of information might be resolved by using information from NDBEMC, and thus it remains the next challenge.
